# Immediate effects of hip strap and foot orthoses on self-reported measures and lower limb kinematics during functional tasks in individuals with patellofemoral osteoarthritis: protocol for a randomised crossover clinical trial

**DOI:** 10.1186/s13063-022-06676-0

**Published:** 2022-09-05

**Authors:** Larissa Rodrigues Souto, Paula Regina Mendes da Silva Serrão, Giulia Keppe Pisani, Bruna Mariana Tessarin, Hygor Ferreira da Silva, Eliane de Morais Machado, Tatiana de Oliveira Sato, Fábio Viadanna Serrão

**Affiliations:** Department of Physical Therapy, Federal University of São Carlos, Rod. Washington Luís, Km 235, São Carlos, SP CEP 13565-905 Brazil

**Keywords:** Gait analysis, Walking, Patellofemoral joint, Hip, Function, Treatment, Rehabilitation, Foot orthoses, Biomechanics, Physiotherapy

## Abstract

**Background:**

Elevated patellofemoral joint stress has been associated with patellofemoral osteoarthritis (PFOA). Changes in lower limb kinematics, such as excessive femoral adduction and internal rotation and excessive rearfoot eversion during the stance phase of functional activities, may increase patellofemoral stress. There is a lack of studies that assess the effects of interventions for controlling femur and subtalar joint movements during functional activities on self-reported measures in individuals with PFOA. Thus, the primary aim of the study is to determine the immediate effects of the hip strap and foot orthoses during level-ground walking and the single-leg squat test on self-reported outcomes. The secondary aim is to investigate whether the hip strap and foot orthoses result in the kinematic changes that these devices are purported to cause.

**Methods:**

Twenty-nine individuals with PFOA aged 50 years or older will take part in the study. The main outcome is pain intensity. The secondary outcomes are other self-reported measures (global rating of change, acceptable state of symptoms, ease of performance, and confidence) and lower limb kinematics (peak femoral adduction and internal rotation, and peak rearfoot eversion). These outcomes will be assessed during functional tasks performed under three conditions: (i) control condition, (ii) hip strap intervention, and (iii) foot orthoses intervention. To investigate whether these interventions result in the lower limb kinematic changes that they are purported to cause, three-dimensional kinematics of the femur and rearfoot will be captured during each task. Linear mixed models with two fixed factors will be used to test associations between the interventions (control, hip strap, and foot orthoses) and conditions (level-ground walking and single-leg squat test) as well as interactions between the interventions and conditions.

**Discussion:**

To the best of the authors’ knowledge, this is the first study to evaluate the immediate effects of the hip strap and foot orthoses on self-reported measures and lower limb kinematics during functional tasks in individuals with PFOA. The findings of this study will enable future trials to investigate the effects of these interventions in rehabilitation programmes.

**Trial registration:**

ClinicalTrials.gov NCT04332900. Registered on 3 April 2020.

**Supplementary Information:**

The online version contains supplementary material available at 10.1186/s13063-022-06676-0.

## Background

Knee osteoarthritis is one of the leading causes of pain and functional disability throughout the world [1]. The most common and symptomatic compartment affected by knee osteoarthritis is the patellofemoral joint [2, 3]. In populations with knee pain or symptomatic knee osteoarthritis, the prevalence of patellofemoral osteoarthritis (PFOA) is 43% [4]. Thus, PFOA constitutes an important source of symptoms and has the potential to exert a negative impact on quality of life due to lifestyle restrictions resulting from pain and functional impairment [5]. The patellofemoral joint is often the first knee compartment affected by osteoarthritis and PFOA is associated with a 5.8-fold greater risk of damage in the tibiofemoral compartment [6]. Thus, PFOA should be considered the focus for the early treatment of knee osteoarthritis [7].

One of the classic symptoms of PFOA is pain in the anterior region of the knee, which is exacerbated by weight-bearing activities that place strain on the patellofemoral joint, such as kneeling and squatting [8]. An altered magnitude or distribution of loading in the patellofemoral joint has been associated with PFOA [8]. Changes in lower limb kinematics, such as excessive femoral adduction and internal rotation during weight-bearing activities, alter the contact between the patella and femoral trochlea, resulting in elevated patellofemoral joint stress [9]. Moreover, excessive subtalar joint pronation (assessed through rearfoot eversion) during the stance phase of gait may result in excessive femur internal rotation and, consequently, result in increased patellofemoral stress [10].

Two studies [11, 12] found also abnormal hip kinematics during functional activities in individuals with PFOA. Crossley et al. [11] found that such individuals walk with greater hip adduction during the late stance phase compared to healthy controls. Carvalho et al. [12] found that individuals with PFOA presented excessive hip adduction at 45° and 60° of knee flexion in both descending and ascending phases of the single-leg squat in comparison to healthy controls. To the best of the authors’ knowledge, only one study [13] investigated foot and ankle characteristics in individuals with PFOA, finding a decrease in ankle dorsiflexion and greater midfoot mobility through clinical measures. These changes may be associated with excessive rearfoot eversion during the stance phase of functional tasks in this population.

As changes in lower limb kinematics observed in individuals with PFOA may increase patellofemoral stress and result in an increase in clinical symptoms, interventions that control excessive femur and rearfoot movements could have an effect on symptoms. A hip strap consists of thin elastic material used to control movements of femoral adduction and internal rotation [14]. Previous studies have demonstrated that a hip strap significantly decreases pain intensity in individuals with patellofemoral pain (PFP) during running, the single-leg squat, and the step-landing task [15–17]. However, to the best of the authors’ knowledge, no studies have evaluated the effect of the hip strap on pain in individuals with PFOA. Foot orthoses with a medial wedge are used to control subtalar joint pronation and, consequently, decrease internal rotation of the femur [18]. Despite strong evidence of foot orthoses decreasing pain intensity in the short term in individuals with PFP [19–21], the effects on clinical symptoms in individuals with PFOA are conflicting [22–25]. To the best of the authors’ knowledge, this will be the first study to evaluate the immediate effects of the hip strap and foot orthoses on self-reported measures and lower limb kinematics during functional tasks in individuals with PFOA. The findings of this study are expected to assist future trials in investigating the effects of these interventions in rehabilitation programmes.

## Methods/design

### Aims

The primary aim of the proposed study is to determine the immediate effects of the hip strap and foot orthoses during level-ground walking and the single-leg squat test on self-reported outcomes. The secondary aim is to investigate whether both the hip strap and foot orthoses result in the kinematic changes that they are purported to cause.

The hypothesis is that the hip strap and foot orthoses will result in immediate improvements in pain intensity and other self-reported measures. We also hypothesise that the interventions will result in a decrease in peak femoral adduction and internal rotation as well as a decrease in peak rearfoot eversion.

### Study design and setting

A within-subject randomised crossover study will be conducted. This is the original version of the study protocol, which has been submitted to the *Clinical Trials* registry (clinicaltrials.gov) and was registered on 3 April 2020 under identification code NCT04332900.

The research will be conducted at the Evaluation and Intervention in Orthopaedics Laboratory (LAIOT) of the Physical Therapy Department of the Federal University of São Carlos (UFSCar) at a controlled temperature (21 to 23 °C).

The following methodology strictly follows the Standard Protocol Items: Recommendations for Interventional Trials 2013 checklist (SPIRIT) [26] and Template for Intervention Description and Replication (TIDieR) [27] to improve the information and quality of intervention reporting [28]. Additional File 1 presents the SPIRIT checklist. Consolidated Standards of Reporting Trials (CONSORT) [29] will be followed for reporting the results in a subsequent article.

### Ethical aspects

This study received approval from the Human Research Ethics Committee of the Federal University of São Carlos (UFSCar), SP, Brazil (certificate number: 24652419.0.0000.5504). The participants will receive clarifications regarding the procedures that will be performed throughout the study and will agree to participate by signing a statement of informed consent. The study will be conducted in accordance with the norms governing research involving human subjects stipulated in Resolution 466/12 of the National Health Board.

### Randomisation and masking

The participants will perform the tasks under three conditions (control condition, hip strap intervention, and foot orthoses intervention) in random order, which will be defined by a random number generator programme (www.randomization.com). Allocation concealment will be achieved using sequentially numbered opaque sealed envelopes. The order will be disclosed until the participants sign the statement of informed consent. A researcher not involved in the assessment or data collection processes will perform the randomisation step. This researcher also will be responsible for obtaining signed informed consent from the participants.

Due to the nature of the interventions administered in this study, the participants, the assessor who will administer the interventions, and the assessor who will conduct the data collection will not be blinded. Blinding will only be performed during the data processing and statistical analysis steps. The researcher conducting these steps will be blinded to the condition to which each participant was assigned. Thus, unblinding will not occur.

### Recruitment

Individuals from the city of São Carlos in the state of São Paulo, Brazil, will be invited to participate in the study. Advertisements for recruitment will be distributed at a university, printed in local newspapers, and posted on social networking websites. An assessor will perform a preliminary screening. An appointment will be scheduled with potentially eligible individuals who agree to participate to confirm eligibility.

### Sample

The R software was used to determine the sample size. Linear mixed model analysis was used to perform the calculation (package sjstats version 0.18.1), power = 80%; *α* = 0.05; large effect size of 1.25; number of clusters = six (three groups × two conditions). Using these parameters, the minimum sample size is 26 participants, to which 10% will be added to compensate for possible dropouts, leading to a sample of 29 participants.

The inclusion criterion is a clinical diagnosis of PFOA [30] adapted from the National Institute for Health and Care Excellence (NICE) guidelines [31]. The following criteria will also be required for inclusion: (i) age 50 years or over; (ii) anterior or retropatellar knee pain aggravated by at least two activities that place strain on the patellofemoral joint (e.g. squatting and stair ambulation); (iii) pain during these activities on most days in the previous month; (iv) pain severity during aggravating activities of ≥ 3 on an 11-point numerical rating scale; (v) symptoms present for at least three months; and (vi) no morning joint stiffness lasting longer than 30 min. The participants will need to have the ability to perform a single-leg squat to at least 60° of knee flexion and have a body mass index between 25 and 34.9 kg/m^2^.

The exclusion criteria are as follows: (i) recent treatment (e.g. knee injections within the previous 3 months); (ii) history of hip, knee, or foot surgery; (iii) physical inability to undergo the testing procedures; (iv) concomitant pain in other knee structures (including the tibiofemoral joint), hip, or lumbar spine; (v) history of knee or hip arthroplasty/osteotomy; (vi) neurological or systemic arthritis; (vii) use of a cane or other gait-assistance device; (viii) history of patellar fracture or recurrent subluxation; and (ix) score on the Mini Mental State Examination (MMSE) suggestive of dementia, taking into account the participant’s educational level.

### Procedures

The participants will undergo a single session. At the beginning of the session, information will be collected on age, duration of symptoms, level of kinesiophoebia determined using the Tampa Scale of Kinesiophobia [32], usual and worst levels of pain experienced in the previous week using the visual analogue scale (VAS) [33], and symptoms related to the knee using the Anterior Knee Pain Scale (AKPS) [34] and Knee Injury and Osteoarthritis Outcome Score (KOOS-BR) [35]. Anthropometric and demographic data will also be collected for the characterisation of the study population. The MMSE [36] will be used to assess cognitive function and the ability to participate in the study. Cut-off points will be determined taking into account the educational level of each participant [36].

**Fig. 1:**
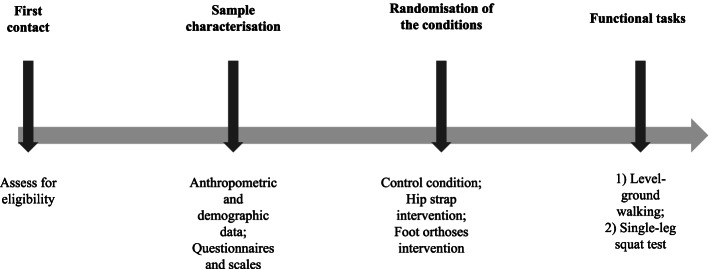
Study design

The participants will be assessed during level walking at a self-selected pace and the single-leg squat test under the three conditions, the order of which will be randomised: (i) control condition, (ii) hip strap intervention, and (iii) foot orthoses intervention. The control refers to the condition in which no interventions will be administered. The design of the study is shown in Fig. 1.

The assessment will be performed on the affected lower limb in cases of unilateral PFOA or on the more painful knee in cases of bilateral PFOA [24]. If the participant reports equal pain intensity in both lower limbs, the lower limb to be tested will be chosen randomly using a simple raffle. For all evaluations, the participants will wear shorts, sports tops (in the case of women), and the same type of shoes (Nike® model *Flex Experience RN 2 MSL*), which will be provided to them by the assessors. The primary and secondary outcomes will be investigated during the performance of two functional tasks: level-ground walking and single-leg squat test.

Data collection and the therapeutic implementation steps will be performed by different assessors during the entire course of the study. Prior to data collection, the participants will have a period of familiarisation with both functional tasks and both therapeutic approaches that will be applied. The participants will perform the two functional tasks in a standardised order. The first will be level-ground walking at a self-selected pace and second will be the single-leg squat test, which is associated with a progressively larger patellofemoral joint load.

The presence of any pain or disability that may be related to the interventions and/or functional tasks will be considered as the criterion to discontinue the study.

### Interventions

#### Hip strap

The hip strap (*S.E.R.F. strap; DonJoy Orthopedics, Inc., Vista, CA, USA*) will be used with the aim of controlling femoral adduction and internal rotation. The hip strap consists of thin elastic material secured to the proximal portion of the leg that wraps in a spiral fashion around the thigh and is anchored around the pelvis. The line of action of the hip strap pulls the femur into abduction and external rotation [14].

#### Foot orthoses

Foot orthoses will be used with the aim of controlling rearfoot eversion. For such, the participants will use a pair of foot orthoses with semi-rigid arch support and medial elevation of 7° at both the forefoot and rearfoot (Propulsão Produtos Biomecânicos, Minas Gerais, Brazil). The foot orthoses will be made from a block of ethyl vinyl acetate with a thermo-mouldable polymer (shore hardness of 45 A) and will be manufactured by an automated computer numeric control machine (CNC routers) [18].

### Functional tasks

#### Level-ground walking

The participants will perform level walking at a self-selected pace on a 7.3-m walkway. The stance phase during level walking will be determined using an AMTI force plate (model OPT400600HF-2000) embedded in the centre of the walkway and synchronised with the three-dimensional motion analysis system with a sampling rate of 1200 Hz. The walkway will be covered with a rubberised fabric so that the participants will be unaware of the force plate and will consequently not alter their walking pattern. Each participant’s walking speed will be calculated by measuring the average horizontal velocity of the marker mounted on the posterior portion of the pelvis [37]. A trial will be considered valid if the foot of the evaluated lower limb comes into full contact with the force plate during the stance phase of gait [23]. If a trial is not considered valid, an additional trial will be performed. The data from the five trials of level-ground walking at a self-selected pace will be collected for analysis. A 1-min interval between trials will be respected.

#### Single-leg squat test

To perform the single-leg squat test, the participants will stand on the lower limb being evaluated and be instructed to squat more than 60° of knee flexion (downward phase of the manoeuvre—2-s period) and return to the starting position (upward phase of the manoeuvre—2-s period) [12, 38]. A digital metronome will be used to control the single-leg squat rate (15 beats per minute). A repetition will be considered valid when the participant performs the single-leg squat with knee flexion of at least 60° within a period of 4 s without losing balance [12, 38]. If a repetition is not considered valid, an additional repetition will be performed. The data from five trials of the single-leg squat test will be collected for analysis. A 1-min interval between trials will be respected [39].

### Primary and secondary outcome measures and assessment points

**Fig. 2:**
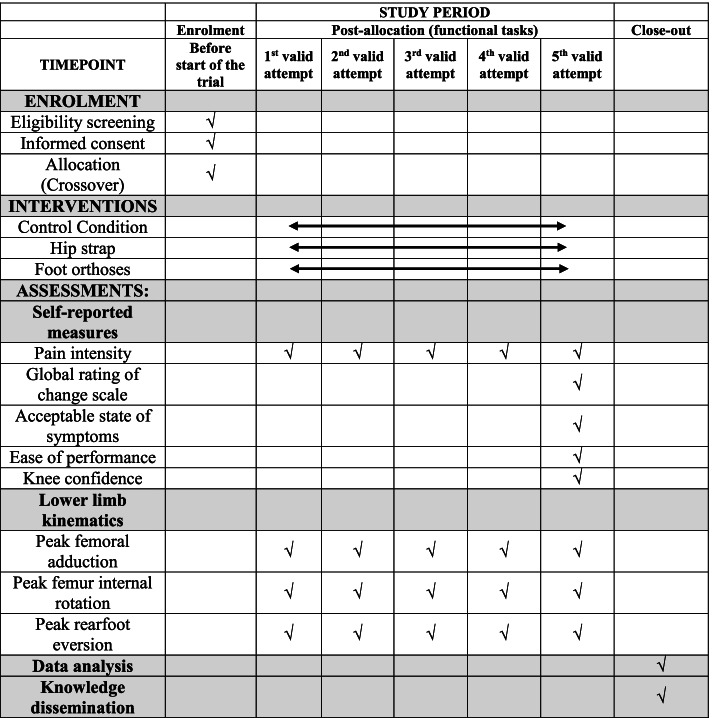
SPIRIT figure of study

The primary outcome will be pain intensity. The secondary outcomes will be other self-reported measures and lower limb kinematic variables, as described below. The data will be collected at a specific time point (Fig. 2). All outcomes will be evaluated by the same assessor.

#### Primary outcome

##### Pain intensity

Pain intensity will be measured using the visual analogue scale (VAS). This scale ranges from 0 to 100 mm, with the leftward extreme marked zero (absence of pain) and the rightward extreme marked 100 (worst pain imaginable). VAS scores will be computed by measuring the length of the line in millimetres from the extreme leftward mark (no pain) to the mark made by the participant. For the statistical purposes, the average score of the five valid attempts of each functional task will be used [33].

#### Secondary outcomes

##### Global rating of change (GRC) scale

The patient’s perception of improvement or deterioration will be quantified using a GRC scale, which is a 15-point Likert-type scale that measures the patient’s impression of a change in health status following a specific treatment [40]. The scale ranges from − 7 (great deal worse) to + 7 (great deal better), with 0 indicating no change. Changes of 4 points or more on this scale have been previously considered clinically important in patients with knee pain [41, 42]. This scale will be applied for both conditions (hip strap and foot orthoses).

##### Acceptable state of symptoms

The acceptable state of symptoms will be assessed by asking the participants the following question: “Considering your usual level of pain and your functional disability to perform daily tasks, if you performed this functional task (level-ground walking or single-leg squat test) with the approach used in this intervention (hip strap or foot orthoses) from now on, would you consider your current state to be satisfactory?” [43]. The participants will answer “yes” or “no”. The question will be posed for both conditions (hip strap and foot orthoses).

##### Ease of performance

To assess the ease of performance during each task, the participants will use a 5-point Likert scale to rate how easy each test was to perform: (i) markedly hard, (ii) somewhat hard, (iii) neither hard nor easy, (iv) somewhat easy, or (v) markedly easy [22].

##### Knee confidence

Knee confidence will be measured using a 100-mm VAS with the following question: “How confident did you feel completing that task?” (terminal descriptors: 0 mm = very confident; 100 mm = not confident at all) [24].

##### Lower limb kinematics

The kinematic variables of interest will be peak femoral adduction and internal rotation and peak rearfoot eversion.

##### Kinematic analysis

Kinematic analysis will be performed using a three-dimensional motion analysis system (Vicon Motion Systems Ltd., Oxford, UK) with six cameras (sampling rate of 120 Hz) during the level-ground walking and single-leg squat tests. Data will be acquired using the Nexus Systems 2.1.1. software (Vicon Motion Systems Ltd., Oxford, UK) and 3D Motion Monitor Software (Innovative Sports Training, Chicago, IL, USA).

**Fig. 3:**
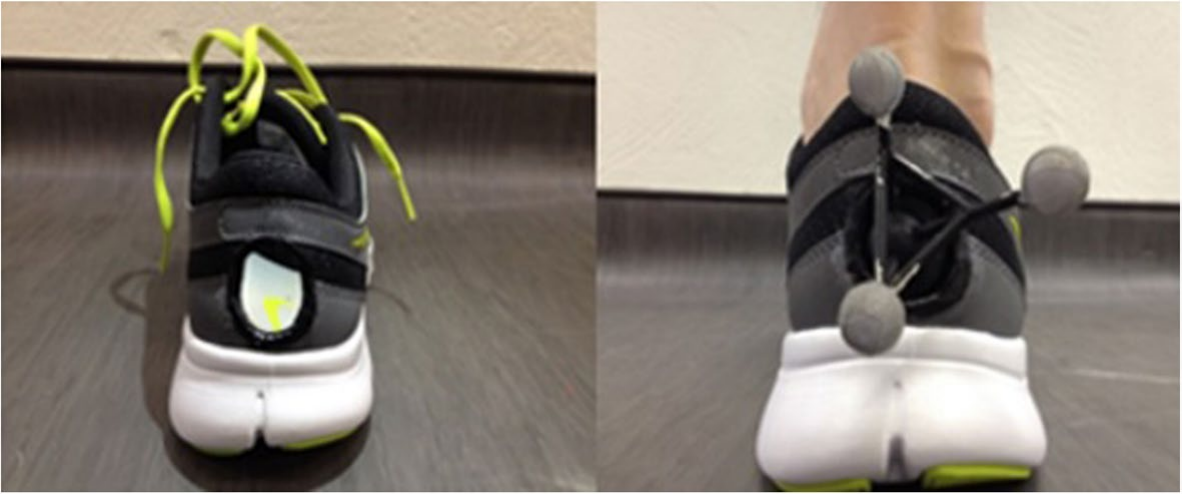
Customised neutral shoes (Nike® model *Flex Experience RN 2 MSL*) with opening in posterior region to allow cluster to be attached directly to calcaneus for kinematic analysis

After the system is calibrated, reflective markers (14 mm in diameter) will be positioned on the following anatomic landmarks: greater trochanter, medial and lateral femoral condyles, medial and lateral malleoli, and base of the fifth metatarsal (on the shoe). Two tracking markers (clusters) consisting of four non-collinear markers will be fixed to a rigid base and attached to the posterolateral portion of the thigh and lower leg using velcro straps. Another cluster consisting of three non-collinear markers fixed to a rigid base will be attached to the rearfoot directly on the calcaneus using double-sided adhesive tape. For this step, the shoes will be customised; an opening in the posterior region will be made to enable the cluster to be attached directly to the calcaneus (Fig. 3). Previous studies have shown that the placement of calcaneal markers on shoes may overestimate the movements of the rearfoot [44]. A static standing trial will be performed to align the subject with global coordinates and provide a reference for further analysis.

##### Kinematic data reduction

Kinematic data reduction will be performed with the 3D Motion Monitor software, which will be used for the creation of a biomechanical model of the body segments. Euler angles relative to the static standing trial will be calculated using the joint coordinate system recommendations of the International Society of Biomechanics [45]. Hip joint centre will be determined using the method proposed by Bell et al. [46, 47]. Ankle joint centre will be defined as the midpoint between the medial and lateral malleoli. The kinematic data will be filtered using a 4th-order, zero-lag, low-pass Butterworth filter with a cut-off frequency of 12 Hz.

Femur and rearfoot motion will be calculated relative to the laboratory coordinate system. The kinematic data will be analysed during the stance phase of level walking as well as during the descending and ascending phases of the single-leg squat test. The stance phase of level walking will be defined as the period between initial contact and toe-off. Using a custom MATLAB (MathWorks Inc., Natick, USA) algorithm, initial contact with the force plate will be considered the moment when the vertical ground reaction force (vGRF) first exceeds 10 N and toe-off will be considered the moment when the vGRF falls below a threshold of 5 N [48].

### Data management

The data will be stored in the UFSCar Physical Therapy Department on a secure computer server with password-protected file, to which only the researchers will have access. Files with the participants’ information will be coded with individual identification codes. The first author will have a backup copy of all data.

### Data monitoring

The researchers involved in the study will be responsible for the monitoring of the protocol, along with any relevant changes that may occur during the development of the study. The Postgraduate Program of the university will supervise the integrity of the data, and the Internal Data Monitoring Committee will have access to the condition to which each participant was assigned, while the whole analysis will be confidential.

### Harms

Adverse events will be collected after the individuals have provided consent and enrolled in the study. Harm and complications from the interventions, if any, will be reported when reporting the results of this trial. Harm will be categorised as serious and minor adverse events.

### Provisions for post-trial care

The first author will be responsible for any injury that occurs during the assessment session and will sign a document for each participant attesting to her commitment to provide any medical treatment, if necessary, in coordination with the Physical Therapy Department at UFSCar.

### Auditing

The Postgraduate Program of the university will supervise the integrity of the data.

### Plans for communicating important protocol amendments to relevant parties

Any modification of the research procedures, including changes in the study objectives, study design, sample size, study procedures, or significant administrative aspects, will lead to a change in the study protocol. This will be under the supervision of the responsible professor (FVS) and approved by the Ethics Committee of the Federal University of São Carlos.

### Statistical analysis plan

Statistical analysis will be conducted with the aid of SPSS (version 22; SPSS Inc., Chicago, IL, USA). The normality and homoscedasticity of the data will be tested using the Shapiro–Wilk and Mauchly tests, respectively. If not normally distributed, data will be transformed to enable the use of parametric tests. All data will be expressed as mean and standard deviation.

Linear mixed models with two fixed factors will be applied to test associations between interventions (control, hip strap, and foot orthoses) and conditions (level-ground walking and single-leg squat test) as well as interactions between interventions and conditions. Linear mixed models were chosen due to the ability to include fixed and random effects and increase the study power by including individuals with missing data in the analysis. Subject and intercept will be included as random effects. The covariance type will be unstructured and the restricted maximum likelihood (REML) estimation method was chosen. When an interaction is significant, the mean difference (MD), standard error (SE), and *P*-value for the pairwise comparisons based on estimated marginal means will be reported. For all analyses, the significance level will be 0.05. Measures such as the 95% confidence interval (CI) and effect size will also be included. Interim analyses will not be performed. No additional analyses will be performed in the trial.

## Dissemination plans

This research is a part of a PhD thesis, the results of which will be disseminated through presentations at conferences, such as regional and national science education conferences, and through articles published in peer-reviewed journals.

## Discussion

PFOA is an important source of knee symptoms and has the potential to exert a negative impact on quality of life due to pain and functional impairment [5]. An altered magnitude or distribution of joint loading in the patellofemoral joint has been associated with PFOA [8]. Changes in lower limb kinematics related to hip adduction [11, 12] as well as clinical changes related to decreased ankle dorsiflexion and greater midfoot mobility [13] observed in this population could result in elevated patellofemoral joint stress during weight-bearing activities. Thus, investigating whether the hip strap and foot orthoses are capable of improving self-reported measures during weight-bearing activities and whether these interventions enable the biomechanical changes that they are purported to do could be important in rehabilitation settings.

## Trial status

This is the original protocol version dated from 3 April 2020. Recruitment has not started. We predict that participants will be recruited between September and November 2022. Study completion is expected to be March 2023.

## Supplementary Information

**Additional file 1.**

## Data Availability

Not applicable, as this manuscript is a protocol and does not contain any data. Any data required to support the protocol can be provided upon request.

## Abbreviations

AKPS: Anterior Knee Pain Scale
KOOS-BR: Knee Injury and Osteoarthritis Outcome Score
CI: Confidence interval
CNC: Computer numeric control machine
CONSORT: Consolidated Standards of Reporting Trials
GRC: Global rating of change
LAIOT: Evaluation and Intervention in Orthopaedics Laboratory
MD: Mean difference
MMSE: Mini Mental State Examination
PFOA: Patellofemoral osteoarthritis
PFP: Patellofemoral pain
REML: Restricted maximum likelihood
SE: Standard error
SPIRIT: Standard Protocol Items: Recommendations for Interventional Trials
TIDieR: Template for Intervention Description and Replication
UFSCar: Federal University of São Carlos
VAS: Visual analogue scale
vGRF: Vertical ground reaction force
